# Portuguese cohort: raltegravir with optimized background therapy (OBT) in multiple-experienced HIV1- and HIV2-infected patients

**DOI:** 10.1186/1758-2652-13-S4-P34

**Published:** 2010-11-08

**Authors:** M Doroana, C Piñeiro, F Maltez, P Fonseca, J Oliveira, K Mansinho, A Horta, E Teófilo, M Aguas, I Germano, D Faria

**Affiliations:** 1 HSM, infectious disease department, Lisboa, Portugal; 2 HSJ, infectious disease department, Porto, Portugal; 3 HCC, medicine department, Lisboa, Portugal; 4 HDF, medicine department, Faro, Portugal; 5 HUC, infectious diseases department, Coimbra, Portugal; 6 HEM, infectious diseases department, Lisboa, Portugal; 7 HJU, infectious diseases department, Porto, Portugal; 8 CHCL, medicine department, Lisboa, Portugal; 9 HGO, infectious diseases department, Almada, Portugal; 10 HSJ, medicine department, Lisboa, Portugal;; 11 CHB, m, Portimão, Portugal

## Purpose

The efficacy and safety profile of raltegravir in the clinical setting were evaluated retrospectively in HIV Portuguese pts treated with raltegravir since the implementation of Early Access and Compassionate Use Program.

## Methods

Pts from 11 Hospitals were enrolled between Mar07-Dec08.Three different subgroups of pts were analyzed (Group1-Pts multi-experienced HIV-1 at virologic failure, Group2-Pts virologically suppressed who needed to change ARV due to toxicity, including T20 replacement and Group3-HIV2 infected pts with failing therapy).OBT was selected based on previous resistance test and prior treatment history. Demographics, co-infections, no. of previous ARV regimens, OBT, adverse reactions and discontinuations were analysed. Immunologic and virological responses were evaluated at baseline, weeks 24 and 48. The primary efficacy endpoint was the proportion of pts with RNA<50 cop/mL and change in TCD4 at weeks 24 and 48. Statistical analysis was performed by SPSS®v18.0.

## Results

A total of 151 pts were eligible for the analysis (107 in Group1, 24 in Group2 and 20 in Group3), 76% were male with a mean age of 47 years, median TCD4 count of 180.0 cells and RNA of 4.3 log10cop/mL. Fifty-one (34%) pts were HCV/HBV co-infected. Median no. of previous ARV treatments was 5.The proportion of pts with RNA<50 cop/mL at week24 (week48) was 70% (69%) in Group1, 100% (100%) in Group2 and 85% (80%) in Group3.Overall median increase in TCD4 count at week24 was 72.0 cells (83.5 in Group1, 31.5 in Group2 and 66.0 in Group3) and at week48 was 99.0 cells (124.5 in Group1, 63.0 in Group2 and 50.0 in Group3).In Group1, 80 pts had PIs in OBT and 76% of these obtained RNA<50 cop/mL vs. 48% without PIs (p=0.006), at week48.In Group3, 13 pts had PIs in OBT and 69% of these obtained RNA<50 cop/mL vs. 100% without PIs, at week48.Adverse reactions occurred in 11 pts but none led to discontinuation.18 pts discontinued:13 therapeutic failures, 2 lost follow-up and 3 deaths (not therapy related).In pts with hepatic abnormalities (AST/ALT) co-infected presented a lower percentage of G3 than no co-infected (67% vs. 83%)/(75% vs. 83%).

## Conclusions

In multiple experienced HIV-infected pts with limited treatment options, raltegravir+OBT has good efficacy as demonstrated in obtaining RNA<50 cop/mL and increasing median TCD4 cell count with a "clean" safety profile namely in co-infected pts. Contrary to HIV1 pts, in HIV2 pts the inclusion of PIs in OBT did not reveal better efficacy.

